# Selective facet etching enables dendrite-less molten salt aluminum metal batteries

**DOI:** 10.1093/nsr/nwaf233

**Published:** 2025-06-03

**Authors:** Meng Zhang, Xin Tong, Lujun Zhu, Fang Liu, Yongfeng Jia, Zhitong Xiao, Daohang Fu, Kang Han, Yu Wang, Hao Zhang, Xuanpeng Wang, Jiashen Meng, Quanquan Pang

**Affiliations:** State Key Laboratory of Advanced Technology for Materials Synthesis and Processing, School of Materials Science and Engineering, Wuhan University of Technology, Wuhan 430070, China; State Key Laboratory of Advanced Technology for Materials Synthesis and Processing, School of Materials Science and Engineering, Wuhan University of Technology, Wuhan 430070, China; State Key Laboratory of Advanced Waterproof Materials, School of Materials Science and Engineering, Peking University, Beijing 100871, China; State Key Laboratory of Advanced Technology for Materials Synthesis and Processing, School of Materials Science and Engineering, Wuhan University of Technology, Wuhan 430070, China; State Key Laboratory of Advanced Waterproof Materials, School of Materials Science and Engineering, Peking University, Beijing 100871, China; State Key Laboratory of Advanced Waterproof Materials, School of Materials Science and Engineering, Peking University, Beijing 100871, China; Hubei Longzhong Laboratory, Wuhan University of Technology (Xiangyang Demonstration Zone), Xiangyang 441000, China; State Key Laboratory of Advanced Technology for Materials Synthesis and Processing, School of Materials Science and Engineering, Wuhan University of Technology, Wuhan 430070, China; Hubei Longzhong Laboratory, Wuhan University of Technology (Xiangyang Demonstration Zone), Xiangyang 441000, China; State Key Laboratory of Advanced Technology for Materials Synthesis and Processing, School of Materials Science and Engineering, Wuhan University of Technology, Wuhan 430070, China; State Key Laboratory of Advanced Technology for Materials Synthesis and Processing, School of Materials Science and Engineering, Wuhan University of Technology, Wuhan 430070, China; Department of Physical Science & Technology, School of Physics and Mechanics, Wuhan University of Technology, Wuhan 430070, China; Hubei Longzhong Laboratory, Wuhan University of Technology (Xiangyang Demonstration Zone), Xiangyang 441000, China; Zhongyu Feima New Material Technology Innovation Center (Zhengzhou) Co., Ltd., Zhengzhou 450001, China; State Key Laboratory of Advanced Technology for Materials Synthesis and Processing, School of Materials Science and Engineering, Wuhan University of Technology, Wuhan 430070, China; Hubei Longzhong Laboratory, Wuhan University of Technology (Xiangyang Demonstration Zone), Xiangyang 441000, China; Zhongyu Feima New Material Technology Innovation Center (Zhengzhou) Co., Ltd., Zhengzhou 450001, China; State Key Laboratory of Advanced Waterproof Materials, School of Materials Science and Engineering, Peking University, Beijing 100871, China

**Keywords:** aluminum battery, selective facet etching, aluminum metal anode, molten salt electrolyte, dendrite-free behavior

## Abstract

Aluminum metal batteries represent a promising alternative to lithium batteries for large-scale energy storage due to their high theoretical capacity, cost efficiency and improved safety features. However, a significant challenge lies in the polycrystalline surface nature of aluminum foil anodes, which can result in uneven aluminum deposition and increase the risk of a short circuit during cycling. Here we report a selective facet-etching method to construct a 3D porous aluminum anode (3D-Al) featuring near-single exposed (220) plane on the surface, enabling dendrite-free behavior and ultra-long cycling for aluminum batteries. The chemical etching mechanism is elucidated to showcase selective removal of the (111) and (200) planes, while preserving (220), which induces directional aluminum plating along the (220) plane. Further, the resulting 3D-Al anode possesses abundant pores and cavities, which serve as preferential sites for aluminum deposition, alleviating volume expansion and effectively inhibiting dendrite growth. The assembled 3D-Al||graphite battery displays excellent rate capability and an ultralong lifespan over 13 000 cycles at a high rate of 10.0 A g^−1^. This work provides a facile yet effective strategy for suppressing aluminum dendrite formation and establishes a new avenue for advancing high-performance aluminum batteries.

## Teaser

The selectively etched aluminum anode with a quasi-single crystalline surface enables uniform plating of Al3^+^ and significantly extends the cycling stability of molten salt aluminum metal batteries.

## INTRODUCTION

Lithium-ion batteries currently dominate the energy storage market due to their high energy and power densities, which make them ideal for portable electronics and vehicles. However, they are inadequate for large-scale energy storage applications [1–3]. This limitation arises mainly from safety issues, high costs and the restricted availability of lithium resources [4,5]. Consequently, it is essential to explore new generations of energy storage batteries. Rechargeable aqueous solution batteries utilizing multivalent metal ions (Mg^2+^, Ca^2+^, Al^3+^ etc.) (Fig. S1) are emerging as promising alternatives [6–11]. These batteries provide high power density, improved safety, abundant resource availability, environmental advantages and excellent ionic conductivity due to their aqueous electrolytes. Among these options, aluminum-ion batteries are particularly notable for their safety in transportation applications and their feasibility for grid-level energy storage, owing to their high theoretical specific capacity, low cost and resource abundance [12–14]. Additionally, aluminum-ion batteries warrant further investigation due to their enhanced safety during operation at optimal temperatures [15].

Nonetheless, aluminum-ion batteries are still in the early stages of development and encounter a number of challenges [16,17]. A significant issue is the formation of metallic aluminum dendrites during uneven plating and stripping, which can result in poor reversibility, reduced capacity, shorter cycle life and potential short circuits [9,18]. The cause of uneven plating may be due to aluminum primarily being plated on the Al (111), (200) and (220) planes, which causes a tip effect that results in irregular buildup and can trigger a short circuit in the battery [19–22]. In addition, the aluminum surface is susceptible to forming a persistent oxide layer upon exposure to air, which obstructs the transfer of Al^3+^ and electrochemical reactions, further complicating these technical challenges [23–25]. To improve metallic aluminum anodes and control aluminum plating behavior at the electrolyte–anode interface, researchers have conducted extensive investigations into interfacial modulation, electrolyte engineering, separator modifications and structural design [26–29]. Although these methods have achieved some success, they are all mainly achieved by introducing other substances (e.g. Zn–Al alloys etc.), which not only increases the cost of the electrode, but also makes the process complicated. Therefore, it is important to design an aluminum anode material based solely on aluminum itself, which is capable of stable cycling during continuous Al plating/stripping processes and is simple to prepare [30–33]. In addition, according to recent studies, it has been shown that 3D metallic anodes can increase the effective contact area of the electrode with the electrolyte, reducing overpotential for Al^3+^ deposition, uniform Al plating distribution and superior electrochemical performance [34,35]. Furthermore, earlier studies have reported a low-cost, fast-charging molten salt aluminum battery that successfully substitutes the conventional ionic liquid electrolyte with an inexpensive inorganic chloride molten electrolyte [1,36–38]. Notably, this economical molten salt electrolyte exhibits relative stability at elevated temperatures, resisting side reactions with electrode materials and effectively preventing the formation of an inert solid electrolyte interphase (SEI) layer. Additionally, the molten salt electrolyte boasts high ionic conductivity, a broad electrochemical window and excellent thermal stability, particularly at high temperatures, making it highly promising for high-power and high-temperature energy storage applications [36,39,40].

In this work, we present a facile selective facet-etching approach for constructing porous Al anodes without passivation layers, achieving uniform Al plating behavior and improved cycling stability. Specifically, the (111) and (200) planes are effectively removed and the (220) plane is effectively enhanced by the etching reaction of Fe(NO_3_)_3_ with Al under boiling conditions. What's more, through the etching reaction, multilayered multidimensional pore-like structures are generated on the Al metal surface, which can effectively induce the uniform plating of Al. The interfacial wettability and carrier transport paths of 3D porous Al foils are significantly improved, increasing the number of aluminum affinities and nucleation sites while reducing the local current density. Thanks to the advantages of the 3D porous structure, aluminum plating preferentially occurs within the pores. In addition, the addition of molten salt electrolyte improved the reaction kinetics and significantly inhibited the growth of dendrites. Electrochemical measurements demonstrated that the etch-treated aluminum foil could extend plating and stripping duration to 520 h at a current density of 1.0 mA cm^−2^, with a cell capacity of 1.0 mAh cm^−2^. In addition, the assembled 3D-Al||graphite molten salt batteries exhibit low polarization and maintain a long lifetime over more than 13 000 cycles. This porous design and the findings from this research offer a straightforward method to inhibit aluminum dendrite growth and establish a strong foundation for the future development of high-performance aluminum batteries.

## RESULTS AND DISCUSSION

### Synthesis and characterizations of the 3D-Al

**Figure 1.**
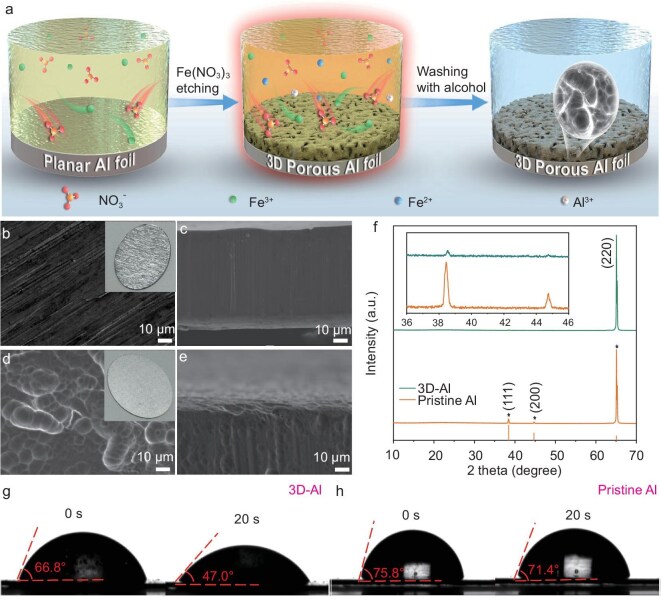
Preparation and characterizations of the 3D-Al. (a) Schematic illustrating the fabrication of 3D porous Al foils in a boiling solution of Fe(NO_3_)_3_. (b, c) Top view and cross-section SEM images of pristine Al and (d, e) 3D-Al (insets are digital photographs of 3D-Al and pristine Al). (f) XRD patterns of 3D-Al and pristine Al foils. (g, h) Comparison of contact angles of the 3D-Al and pristine Al foil.

The preparation of the 3D-Al foil through a boiling Fe(NO_3_)_3_ solution etching process is illustrated in Fig. 1a. Specifically, the aluminum foil is immersed in the Fe (NO_3_)_3_ solution and the mixture is quickly heated to boiling using a heat oven. At the same time, Fe(NO_3_)_3_ solution acts as a strong oxidant under boiling conditions, and undergoes a redox reaction with the surface of the aluminum foil, which improves the surface properties of the aluminum foil [41]. Due to the polycrystalline surface nature of aluminum foil anodes, there are different etching speeds on different crystal surfaces. This selective etching phenomenon causes the formation of a porous structure on the Al surface. In addition, the boiling conditions accelerate this reaction, as the high temperature increases the kinetic energy of the reactant molecules. Bubbles generated from the bottom are more likely to collide and react, facilitating the creation of a porous structure in the aluminum foil immerged in the boiling Fe(NO_3_)_3_ solution. Additionally, the boiling process promotes thorough mixing of the solution, enhancing reaction efficiency.

Unlike the smooth surface and cross-section of conventional 2D planar aluminum foils (Fig. 1b and c), the surface and cross-sectional microstructure of 3D porous aluminum foils (3D-Al) (Fig. 1d and e), which have been etched using a boiling Fe(NO_3_)_3_ solution, are characterized by scanning electron microscopy (SEM). These 3D-Al foils display abundant pores ranging from nanoscale to micrometer diameter distributed across their surface. Moreover, the porous structure is evident in the cross-sectional SEM images, showing random pores of several hundred nanometers uniformly distributed within the aluminum foil, resulting in an interconnected 3D structure. At higher magnifications, the 3D-Al foil is etched to a depth of up to 7 μm (Fig. S2). In addition, in contrast to the surface color of the pristine metallic aluminum foil, the 3D-Al surface exhibits a silvery appearance. It is worth noting that the structure of the holes on the surface of the 3D-Al foil becomes more uniform and the depth of the holes becomes deeper as the concentration of the Fe(NO_3_)_3_ solution grows (Fig. S3) or the reaction time grows (Fig. S4). Analysis of the Al(III) content in the residual precursor solution using full-spectrum direct-reading plasma emission spectroscopy indicates that approximately one-third of Al^3+^ is present in the solution, confirming that the aluminum foil was successfully corroded by the boiling Fe(NO_3_)_3_ solution (Fig. S5). Furthermore, X-ray photoelectron spectroscopy (XPS) analysis of the Fe *2p* spectra demonstrates the presence of Fe(II) and Fe(O) species on the surface of the 3D-Al metal (Fig. S6).

The physical properties of the obtained porous aluminum foil were further examined and confirmed by X-ray diffraction (XRD) to be consistent with the previous results (Fig. 1f). Main lattice (111), (200) and (220) planes were identified in pristine Al metal at 38.5°, 44.7° and 65.1°, with peak intensity ratios of 100:39:1476.3 for pristine aluminum (111), (200) and (220), respectively. In contrast, the 3D-Al foils etched with boiling Fe(NO_3_)_3_ solution had significantly weakened (111) and (200) planes at 38.5° and 44.7°, while the proportion of the (220) plane was notably increased. This resulting near-single plane property is beneficial for uniform aluminum plating/stripping processes. To achieve a uniform aluminum plating surface, it is essential for the aluminum foil to have a hydrophilic surface for molten salt electrolyte infiltration [42]. This was assessed using contact angle measurements. Thanks to its 3D porous structure and chemically modified surface, 3D-Al exhibits superior wettability compared to pristine aluminum foils, with a contact angle of only 47° after 20 s of exposure (Fig. 1g). In comparison, the contact angle of the pristine aluminum foil remained nearly stable at 71.4°, even after 20 s (Fig. 1h). The hydrophilic characteristics of 3D-Al enhance the surface contact with the electrolyte, thereby facilitating uniform aluminum plating through more consistent ion transport. XPS analysis was performed to confirm the chemical state of the Al metal surface. The high-resolution Al *2p* spectrum of pristine aluminum metal primarily displays an Al-metal peak at 71.7 eV and an Al-oxide peak at 74.4 eV. The etched 3D-Al preserves the properties of pristine aluminum remarkably well, showing negligible shifts in the characteristic peaks (Fig. S7) [43,44].

**Figure 2.**
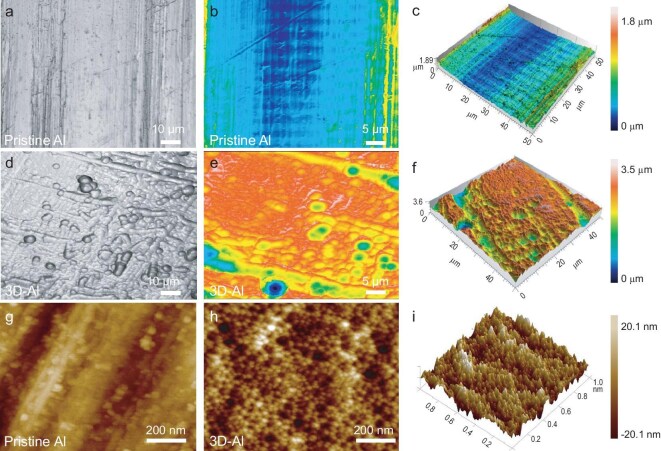
Morphology characterizations of the 3D-Al and pristine Al. (a–f) LSM images of 3D-Al and pristine Al foils. (g–i) AFM images of 3D-Al and raw Al foils (de-noised by Nano Scope analysis software).

To elucidate the surface topography of the resulting 3D-Al foil and pristine Al foil, various characterization techniques were employed, including SEM, infrared spectroscopy combined with an atomic force microscopy (AFM) test system under multiple field actions, and laser co-polymerization scanning microscopy (LSM) [45–47]. First, SEM images at higher magnification clearly reveals nanoscale voids along the sidewalls of the micron-sized voids (Fig. S8). The surface of the pristine aluminum foil was examined using LSM at a magnification of 10 µm, resulting in the construction of a 3D image that shows a relatively flat surface (Fig. 2a–c). In contrast, the surface of 3D-Al exhibits abundant uniform-sized holes, which are more intuitively visible in the 3D image, revealing an internal structure of interconnected voids (Fig. 2d–f). Furthermore, the roughness of 3D-Al appears greater than that of the pristine aluminum foil. The average depth of the holes is approximately 1.93 µm, and the average volume of the holes is 125 µm^3^, providing increased space for aluminum plating (Fig. S9 and Table S1). Additionally, AFM was utilized to quantitatively characterize the surface roughness of the 3D-Al (Fig. 2g–i). The 2D image clearly shows abundant interconnected holes on the surface. This observation confirms the presence of an interconnected porous structure within the scanned image. Importantly, the constructed 3D image indicates uniformly rough pore cavities, with the roughness of 3D-Al being significantly greater than that of pristine aluminum (Fig. S10).

To further demonstrate the feasibility of the etching process, a large area of aluminum foil was etched in a larger vessel (Fig. S11) and the structural uniformity and single crystalline surface of the porous aluminum foil remained, further demonstrating its engineering feasibility (Fig. S12). It is worth noting that in order to further explore the potential of the selective etching strategy, this approach was extended to magnesium and zinc. The structure of magnesium also exhibited reactivity with respect to crystalline facets (Fig. S13), whereas the etching kinetics of zinc may require further optimization due to oxidation tendencies or reaction conditions (Fig. S14).

### Al plating/stripping behavior of the 3D-Al

**Figure 3.**
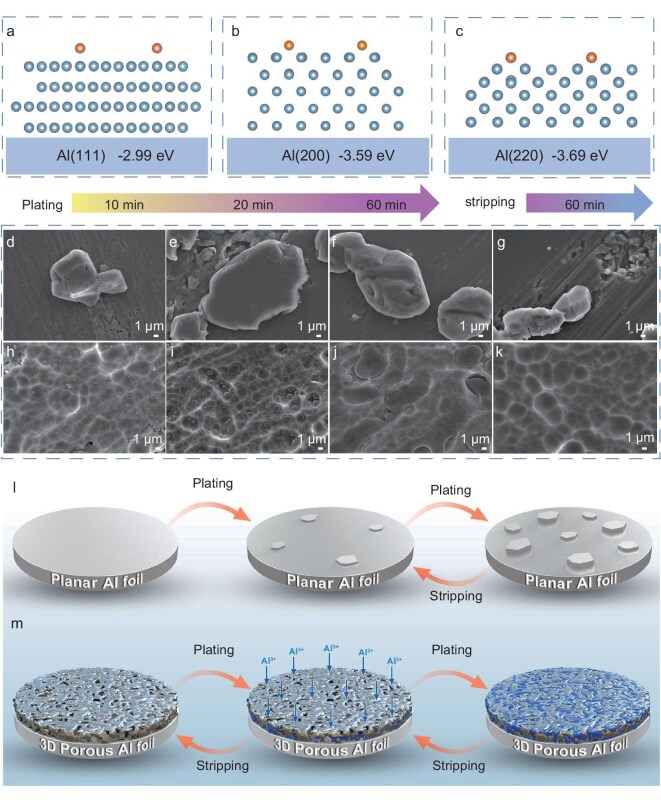
Electrochemical aluminum plating/stripping behaviors in molten salt electrolyte. (a–c) Simulation models of Al atomic attachment on different (111), (200) and (220) planes of Al foil. (d–g) Morphological evolution of pristine Al and (h–k) 3D-Al electrodes during the plating/stripping process with different durations at a current density of 0.5 mA cm^−2^. (l, m) Schematic representation of the proposed plating/stripping process using pristine Al and 3D-Al electrodes.

Previous studies indicate that the number and arrangement of atoms vary across different crystal planes, suggesting that the plating morphology of aluminum differs when using substrates with different preferred crystal plane orientations [48]. Structural models were created to represent the additional adsorbed aluminum atoms on the various crystal planes (Fig. 3a–c). The interaction between aluminum atoms and the Al surface was evaluated based on adsorption energy. The stable adsorption energies for aluminum atoms are measured at −2.99 eV on the (111) plane, −3.59 eV on the (200) plane and −3.69 eV on the (220) plane. The lowest adsorption energies suggest that aluminum has a greater tendency to nucleate on the (220) plane compared to the (200) and (111) planes. Based on this theoretical analysis, to modulate the deposition behavior of aluminum, we eliminated the Al (111) and Al (200) crystal planes and preferentially exposed more Al (220) crystal planes through a boiling Fe(NO_3_)_3_ etching reaction, facilitating aluminum plating along a single crystal plane. This approach aligns with the XRD data presented earlier.

Notably, two regions of the 3D-Al foil were selected for analysis from the grain orientation map of electron backscattered diffraction (EBSD). It can be found that the green grains represent the {110} orientation and the grain orientation is mainly concentrated in the {110} direction (i.e. (220) orientation) (Fig. S15a). By analysing the antipodal map, it was found that the {110} direction has the highest orientation strength of 4.39 (Fig. S15b). We randomly selected a larger area, and the orientation of the grains was also mainly concentrated in the {110} direction (Fig. S15c). The {110} direction also has the highest orientation intensity of 11.35 (Fig. S15d), which indicates that in 3D-Al materials, the orientation of the grains is mainly dominated by the {110} direction. It is further verified that the 3D-Al foil has a significant {110} orientation. This result is in agreement with XRD results. In addition, transmission electron microscopy (TEM) and high-angle annular dark-field scanning transmission electron microscopy (HAADF-STEM) images show that the aluminum sheet consists of multiple single-crystal aluminum particles (Fig. S16a and b). The size of the aluminum particles is about 2 µm. Fast Fourier transform (FFT) images of the labelled regions confirm that the particles are single crystals. The distinctive and intense (110) facets indicate that they are predominantly present in 3D-Al (Fig. S16c). Combined with the HAADF-STEM image and energy dispersive spectroscopy (EDS) mapping image (Fig. S16d–f), it can be seen that the Al elements are uniformly distributed in the 3D-Al foil region.

To observe the plating and stripping behavior of aluminum in the molten salt electrolyte, the morphological evolution of 3D-Al and pristine aluminum electrodes was characterized at various time intervals using *ex situ* SEM. The pristine Al presents numerous large Al protrusions during the initial plating stage. Subsequently, the ongoing accumulation of aluminum at the tips of these protrusions causes dendrite growth and results in an inhomogeneous plating morphology, ultimately leading to cell failure (Fig. 3d–f). As expected, during deposition times ranging from 10 to 60 min, irregular lumps with varying orientations formed on the surface of the pristine aluminum. Even after 60 min of stripping, the protruding aluminum remained on the foil surface (Fig. 3g). In contrast, the deposition on 3D-Al maintained a flat surface throughout its evolution and displayed raindrop-like plating (Fig. 3h–j). Specifically, after 10 min of deposition, aluminum was deposited on the 3D-Al surface as random dots dispersed among the holes, which gradually filled these holes as deposition time increased to 30 and 60 min. This phenomenon is more clearly demonstrated in the cross-section of the 3D-Al (Fig. S17). Additionally, it is confirmed that aluminum plating on the surface of the 3D-Al electrode is self-selectively deposited along the (220) plane. Furthermore, after 60 min of stripping, the 3D structure is clearly reinstated (Fig. 3k).

Based on experimental observations, we conducted COMSOL simulations to model the dynamic process of electrochemical plating and stripping on 3D-Al foils. The 3D porous aluminum model used in COMSOL simulations was constructed based on the experimental SEM images in Fig. S18. The dynamic plating and stripping processes were simulated to visualize the aluminum plating and the variation of aluminum ion concentration along the pore walls. The plating process resulted in a gradual reduction in pore diameter. At the same time, the Al^3+^ distribution tended to be more uniform due to the migration of Al^3+^ into the pores during plating. During the dissolution phase, the pore diameter widens reversibly, while the Al^3+^ concentration near the pore bottom increases (Fig. S19). Consequently, the simulated deposition closely resembles the *ex situ* SEM results, where plating initiates from the bottom region and extends along the pore walls before the pore structure recovers during dissolution. This integrated information serves as strong evidence that 3D-Al electrodes can effectively regulate Al^3+^ reaction kinetics and mitigate dendrite growth. This improvement is mainly attributed to the well-developed graded pores and voids, which offer numerous nucleation sites and modify potential distribution. Correspondingly, the plating and dissolution of 3D-Al and pristine Al within the porous structure are clearly illustrated in Fig. 3l–m. Al deposition can be preferentially plated inside the 3D-Al cavity rather than preferentially on the aluminum plane. Although the deposited layer will gradually increase after a few cycles, aluminum plating will be effectively suppressed in the 3D-Al cavity structure, preventing it from growing upwards and penetrating the diaphragm. In addition, another advantage of the 3D-Al is its ability to enhance transport kinetics. 3D-Al offers rapid pathways for ions and electrons, enhances the effective area of the solid-electrolyte interface, decreases local current density, and buffers volume changes. This effectively inhibits the formation of dead aluminum and mitigates internal polarization diffusion during repeated cycling.

### Electrochemical performance of symmetric cells

**Figure 4.**
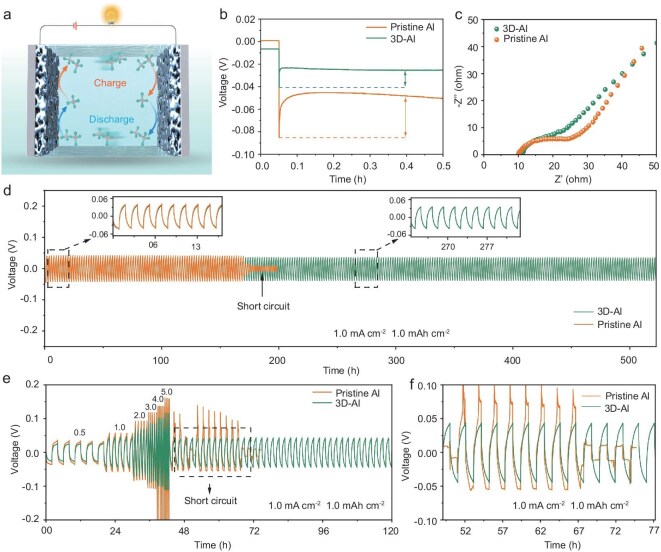
Electrochemical performance of symmetric cells in molten salt electrolytes. (a) Schematic of a symmetric cell assembled with 3D-Al. (b) Voltage profiles of 3D-Al and pristine Al at the first plating at a current density of 1.0 mA cm^−2^ and a capacity of 1.0 mAh cm^−2^. (c) EIS spectra of the initial state of 3D-Al and pristine Al foils. (d) Long-term plating/stripping performance of 3D-Al and pristine Al (inset shows their amplified voltage curves). (e, f) Multiplicity performance of 3D-Al and pristine Al foils at various current densities from 0.5 to 5.0 mA cm^−2^.

Constant current cycling measurements were performed to evaluate the electrochemical performance of 3D-Al||3D-Al symmetric cells (Fig. 4a). The cells were cycled at a fixed current density with a total capacity of 1.0 mAh cm^−2^ and voltage–time curves were used to monitor any instabilities in cells. Specifically, the initial plating voltage profiles of 3D-Al and Al foils at a current density of 0.5 mA cm^−2^ also reflect differences in their electrochemical properties (Fig. 4b). The voltage profile indicates that the nucleation overpotential of the 3D-Al electrode is 14.8 mV, significantly lower than that of the pristine aluminum foil, which is 36.6 mV at 0.5 mA cm^−2^. Notably, the nucleation overpotential of the 3D-Al electrode disappears at a current density of 1.0 mA cm^−2^ (Fig. S20). Furthermore, electrochemical impedance spectroscopy (EIS) was employed to elucidate the charge transfer kinetics of the prepared 3D-Al anodes (Fig. 4c). The 3D-Al-based symmetric cells showed much smaller semicircles compared to pristine Al in the initial state, implying an improvement in the intrinsic conductivity of the 3D-Al electrodes. In addition, the electrochemical surface area of 3D-Al and Al foil was evaluated by cyclic voltammetry (CV) measurements to reveal the active sites for Al plating. The 3D-Al electrode showed a larger electrochemically active surface area compared to the Al foil (Fig. S21). Therefore, the 3D-Al||3D-Al symmetric cell has a longer lifetime and higher stability than the Al||Al symmetric cell at a current density of 1.0 mA cm^−2^. The cycling performance of the 3D-Al||3D-Al symmetric cell reaches 520 h with a cut-off capacity of 1.0 mAh cm^−2^ at 1.0 mA cm^−2^ (Fig. 4d). The post-deposition surface morphology of the 3D-Al and Al foils further demonstrates this difference (Fig. S22), with SEM images of 3D-Al after deposition at a current density of 1.0 mA cm^−2^ showing a more homogeneous plating morphology. This validates the advantages provided by the effective etching strategy employed for the 3D-Al anode and the use of ferric nitrate. Additionally, to further investigate the reaction kinetics, the rate performance of the cells was evaluated by cycling them at various current densities with a fixed plating/stripping capacity of 1.0 mAh cm^−2^. The cell polarization voltages were 47, 80, 125, 157, 184 and 210 mV at current densities of 0.5, 1.0, 2.0, 3.0, 4.0 and 5.0 mA cm^−2^ (Fig. 4e and f). The voltage profile of 3D-Al remained lower than that of Al when the current density was restored to 1.0 mA cm^−2^. This stable performance indicates that the 3D-Al foils, characterized by a 3D layered porous structure, reduce the polarization of the metal anode and lower local current density, facilitating an efficient aluminum plating and stripping process. Meanwhile, the 3D-Al||3D-Al symmetric cell achieves stable cycling with a fixed capacity of 1.0 mAh cm^−2^ when tested at a current density of 5.0 mA cm^−2^, the polarization becoming smaller as the cycling progresses. Notably, the curves exhibit good overlap and display a skewed character as cycling continues, indicating favorable reversibility of the ion storage behavior (Fig. S23). Furthermore, the 3D-Al||3D-Al symmetric cell achieves stable cycling with a fixed capacity of 5.0 mAh cm^−2^ at a current density of 1.0 mA cm^−2^ and decreasing polarization as the cycling progresses (Fig. S24). To investigate the maximum endurable current density of 3D-Al composite and pristine Al metal anode, the critical current density (CCD) was measured in 3D-Al-based and pristine Al-based symmetric cells. As shown in Fig. S25, the CCD value of 3D-Al is 30.0 mA cm^−2^ within a capacity of 1.0 mAh cm^−2^, higher than that of pristine Al (8.0 mA cm^−2^).

### Electrochemical performance of 3D-Al||graphite cells

**Figure 5.**
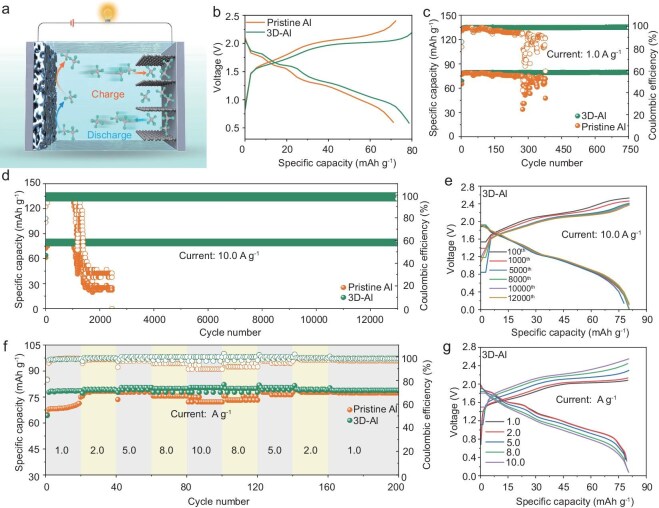
Electrochemical performance of 3D-Al||graphite molten salt battery. (a) Schematic of a 3D-Al||graphite molten salt battery. (b) Charge–discharge curve for the 10th cycle at a current density of 1.0 A g^−1^. (c) Lifetime and stability at a current density of 1.0 A g^−1^. (d) Cycling performance of 3D-Al and pristine Al foils at a current density of 10.0 A g^−1^. (e) Voltage curves of 3D-Al||graphite molten salt battery with different numbers of turns. (f) Rate performance at different current densities. (g) Voltage curves of 3D-Al||graphite molten salt battery with different numbers of turns.

To further evaluate the practical application of 3D-Al, we assembled a 3D-Al||graphite molten salt battery (Fig. 5a). For comparison, a pristine Al||graphite molten salt battery was studied. Typical CV curves of the assembled 3D-Al||graphite molten salt battery are shown in Fig. S26; the 3D-Al||graphite molten salt battery exhibits sharp redox peaks and high peak intensities. AC impedance spectroscopy further validates lower polarization and faster diffusion kinetics of the 3D-Al||graphite molten salt battery (Fig. S27). Additionally, the constant-current charging curves indicate reduced polarization of the 3D-Al electrode. The discharge specific capacities of the 3D-Al||graphite battery and Al||graphite battery in the 10th cycle were 78.6 and 71.1 mAh g^−1^, respectively (Fig. 5b). Both charge and discharge curves exhibit a flat discharge plateau, which is consistent with the observations in the CV curves.

At a current density of 1.0 A g^−1^, the 3D-Al||graphite molten salt battery maintains a high discharge capacity of 78.9 mAh g^−1^ after 750 cycles with almost no capacity degradation (Fig. 5c), indicating excellent cycling stability (Fig. S28). In contrast, the cell with pristine aluminum anode electrodes exhibited inferior cycling stability and lower Coulombic efficiency (30.2%), starting with an initial discharge capacity of 52.8 mAh g^−1^, which dropped to 24 mAh g^−1^ after 300 cycles, highlighting the superior durability of the 3D-Al anodes. At a high rate of 10.0 A g^−1^, the 3D-Al||graphite battery exhibits remarkable cycling stability without capacity decay over 13 000 cycles (Fig. 5d). The different charge/discharge curves of 3D-Al||graphite batteries overlapped, indicating good reversibility (Fig. 5e). In contrast, the pristine Al||graphite battery only survives for about 2000 cycles. This difference may be attributed to the poorer irreversibility of planar aluminum, leading to the formation of large grains. After cycles, SEM images of pristine Al foil clearly show uncontrolled lumpy morphology, whereas the 3D-Al anode shows a flat surface (Fig. S29). Specifically, the 3D-Al||graphite molten salt battery provides specific capacities of 78.6, 79.4, 80.6, 80 and 80.6 mAh g^−1^ at current densities of 1.0, 2.0, 5.0, 8.0 and 10.0 A g^−1^ (Fig. 5f). When the current density was restored to 1.0 A g^−1^, the specific capacity of the 3D-Al||graphite battery remained at 79.4 mAh g^−1^. The results indicate that the 3D-Al electrode exhibits excellent multiplicative performance and cycling stability, showcasing broad application potential. Furthermore, the corresponding charge and discharge curves at varying current densities demonstrate that a distinct plateau characteristic is maintained at each charging rate (up to 10.0 A g^−1^), along with an increase in voltage polarization as current density rises (Fig. 5g). Notably, the performance of the 3D-Al anode is compared to that of other modified aluminum anodes reported in recent studies (Fig. S30) [49–54]. To explore the potential of the 3D-Al in ionic liquids, 3D-Al||graphite and pristine Al||graphite batteries were further assembled using ionic liquids ([EMIm]Cl-AlCl_3_) as the electrolyte. At a current density of 1.0 A g^−1^, the 3D-Al||graphite battery has an initial discharge capacity of 68.98 mAh g^−1^ with 1800 stable cycles with almost no capacity degradation (Fig. S31b and c). On the other hand, the initial discharge capacity of pristine Al was 68.69 mAh g^−1^ and the cell showed short circuit and capacity decay after 360 cycles. The 3D-Al anode in ionic liquid still showed excellent cycling stability. Importantly, symmetric cell tests showed that 3D-Al symmetric cells in ionic liquids still had 192 h of stable cycling at a current density of 1.0 mA cm^−2^, 1.0 mAh cm^−2^ (Fig. S31a). These data indicate that the advantages of 3D-Al's porous structure are versatile in different electrolyte systems, effectively suppressing dendritic growth and improving cycling stability.

### Practical evaluation of 3D-Al||graphite batteries

**Figure 6.**
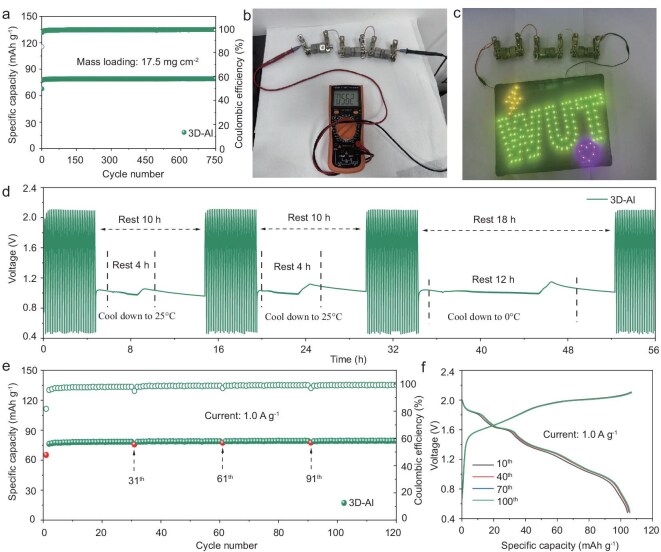
Practical evaluation of the 3D-Al||graphite molten salt battery. (a) Cycling performance and voltage profiles of the graphite with a mass loading of 17.5 mg cm^−2^ at 1.0 A g^−1^ and 90°C. (b) Voltage of 3D-Al||graphite molten salt battery. (c) LED lit by 3D-Al||graphite molten salt battery. (d–f) Freeze–thaw test of 3D-Al||graphite molten salt batteries with multiple terminations during charging and discharging and cooled to 25°C or 0°C.

To further evaluate the practical feasibility of the 3D-Al anode, 3D-Al||graphite molten salt batteries were assembled. Even at 90°C and a graphite loading of 17.5 mg cm^−2^, the battery exhibits a high reversible capacity of 80.0 mAh g^−1^ at a current density of 1.0 A g^−1^, along with excellent cycling stability and no capacity degradation over 750 cycles (Fig. 6a). This demonstrates the high area capacity and near-100% Coulombic efficiency attainable with 3D-Al materials. We further evaluate the potential of the 3D-Al||graphite molten salt battery in practical applications. After the battery was fully charged, its voltage was recorded at 1.837 V (Fig. S32). When three 3D-Al||graphite molten salt batteries were connected in series, the voltage stabilized at 5.62 V (Fig. 6b), enabling the simultaneous illumination of 130 light-emitting diodes (LEDs) (Fig. 6c). Additionally, we conducted freeze–thaw experiments, confirming that this process is essential for efficient long-term energy storage, potentially paving the way for seasonal energy storage and renewable energy integration [55]. Specifically, a molten salt battery electrolyte in a controlled state enables charging and discharging operations to occur solely at high temperatures, where the electrolyte remains in liquid form. When the temperature drops and causes the electrolyte to solidify, the chemical energy stored in the battery can be effectively preserved. After being stored for some time at a current density of 1 A g^−1^ with the oven turned off and allowed to cool to room temperature (or even after prolonged periods at around 0°C), the battery can be reheated to 90°C to melt the solidified electrolyte and continue normal charging and discharging operations (Fig. 6d). Furthermore, the molten salt electrolyte melts each time it is reheated, leading to improved capacity retention and exceptional cycling stability (Fig. 6e). Significantly, the polarization decreased as the cycles progressed after various instances of restarting the oven. Notably, the curves exhibited good overlap and displayed a skewed feature during the 10th to 100th cycles, indicating favorable reversibility in ion storage behavior (Fig. 6f). This phenomenon may be attributed to improved contact between the electrolyte and the electrodes after re-melting.

## CONCLUSION

We present a novel and effective strategy for achieving uniform aluminum plating and stable cycling, enabling the fabrication of multilayered 3D porous aluminum anodes through a boiling iron nitrate treatment. This innovative approach selectively removes the (111) and (200) planes while preserving the (220) plane, facilitating directional aluminum plating along the (220) single crystal orientation. The method generates a hierarchical porous structure, with pore diameters spanning from nanoscale to microscale dimensions on the aluminum surface. This unique architecture significantly enhances electrolyte accessibility, increases nucleation site density, effectively mitigates aluminum dendrite formation and accommodates volume fluctuations during cycling. Compared to conventional planar Al foil, the 3D-Al exhibits substantially increased surface area, which significantly improves charge transfer kinetics for electrochemical reactions. The 3D-Al electrode demonstrates a distinctive low-overpotential bottom-top aluminum deposition behavior during plating/stripping cycles, representing a key advancement in this study. Furthermore, the well-defined pore architecture of the 3D-Al electrodes effectively restricts non-uniform aluminum growth, enabling symmetric cells to maintain stable cycling performance for over 520 h at a current density of 1.0 mA cm^−2^ with a cut-off capacity of 1.0 mAh cm^−2^. The optimized 3D-Al||graphite molten salt batteries exhibit significantly enhanced rate capability and exceptional cycling stability, surpassing 13 000 cycles with minimal capacity degradation. The simplicity and effectiveness of this strategy make it readily applicable to other metal systems, offering a promising pathway for developing stable and high-performance battery technologies.

## METHODS

Detailed materials and methods are available in the online Supplementary data.

## Supplementary Material

nwaf233_Supplemental_File
