# Supplementary material for: Selective facet etching enables dendrite-less molten salt aluminum metal batteries
Source: Natl Sci Rev. 2025 Jun 3;12(7):nwaf233. doi: 10.1093/nsr/nwaf233 (PMC12236164; doi:10.1093/nsr/nwaf233)
Supplement: nwaf233_Supplemental_File [file nwaf233_supplemental_file.pdf]

Supporting Information

Selective facet etching enables dendrite-less molten salt aluminum metal batteries

Meng Zhang[1], Xin Tong[1], Lujun Zhu[2], Fang Liu[1]*, Yongfeng Jia[2], Zhitong Xiao[2], Daohang Fu[4], Kang Han[1,4], Yu Wang[1], Hao Zhang[1], Xuanpeng Wang[3,4,5], Jiashen Meng[1,4,5]*, Quanquan Pang[2]*

1. M. Zhang, X. Tong, K. Han, Y. Wang, H. Zhang, K. Luo, F. Liu, J. Meng

State Key Laboratory of Advanced Technology for Materials Synthesis and Processing, School of Materials Science and Engineering, Wuhan University of Technology, Wuhan 430070, P.R. China

E-mail: fangliu212@whut.edu.cn; jsmeng@whut.edu.cn

2. Lujun Zhu, Yongfeng Jia, Zhitong Xiao, Quanquan Pang

School of Materials Science and Engineering, Peking University, Beijing, 100871, China

E-mail: qqpang@pku.edu.cn

3. X. Wang

Department of Physical Science & Technology, School of Physics and Mechanics, Wuhan University of Technology, Wuhan 430070, P. R. China

4. D. Fu, K. Han, X.Wang, J. Meng

Hubei Longzhong Laboratory, Wuhan University of Technology (Xiangyang Demonstration Zone), Xiangyang 441000, Hubei, China

5. X. Wang, J. Meng

Zhongyu Feima New Material Technology Innovation Center (Zhengzhou) Co., Ltd., Zhengzhou, Henan 450001, China

Section S1 Experimental Procedures

Experimental part

Materials

Al foil (99.99% purity), $Fe(NO_3)_3$ $9H_2O$ (99.99% purity), $AlCl_3$ (99.99% purity), NaCl (99.99% purity), LiCl (99.99% purity), and KCl (99.99% purity) were purchased directly from Aladdin, and natural graphite was purchased from Clorox.

Preparation of the 3D-Al foil

Firstly, Al foils were cut into 10 mm discs, placed into a beaker containing 50 ml of $Fe(NO_3)_3$ (2.5 Mm pH 1.58) solution and boiled in an electronic multifunction oven at 1000 W for 180 s. The $Fe(NO_3)_3$ solution was poured off and washed several times with deionised water. Specifically, the thickness and size of the holes can be determined by repeated boiling. The final resulting porous Al foil is washed several times with ethanol and dried in air, denoted 3D-Al.

Preparation of the molten salt electrolyte

All the following processes were done in an argon filled glove box (conditions: O_2 < 0.01 ppm, H_2O < 0.01 ppm). To prepare the tetrameric molten salt electrolyte, NaCl (Aladdin, 99.9%), LiCl (Aladdin, 99.9%) and KCl (Aladdin, 99.9%) were first heated in a muffle furnace at 300 °C to remove water from the salt. Anhydrous $AlCl_3$, NaCl, LiCl and KCl were weighed and added to a glass weighing flask with a lid in a certain molar ratio (1.3:0.43:0.42:0.15). The weighing flask containing the four inorganic salt components was then sealed and shaken well and heated to 160 °C in a muffle furnace and kept for 48 h to obtain a homogeneous and clear liquid. The clarified liquid was poured into a mortar and pestle while still hot and cooled to room temperature to solidify, and then the resulting solid mixture was thoroughly ground to obtain a white powdered molten salt tetrameric electrolyte.

Ionic liquid electrolyte preparation

Room temperature ionic liquid electrolyte was made by mixing anhydrous aluminum chloride ($AlCl_3$) and 1-ethyl-3-methylimidazolium chloride ([EMIm]Cl) in a molar ratio of 1.3:1. The mixture was then homogeneously stirred and mixed for 24 hours to obtain a pale yellow ionic liquid.

Material characterizations

X-ray diffraction (XRD) patterns of $Fe(NO_3)_3$ solution-etched Al and pristine Al were recorded on a D8 advanced X-ray diffractometer (Bruker, Germany) using Cu Kα radiation (λ = 1.5406 λ). The morphology and thickness of the 3D-Al and Al foils were characterised by using a FESEM (JEOL JSM-7600 F) at 15.0 kV. X-ray photoelectron spectroscopy (XPS) analyses were performed on a Kratos Axis Supra photoelectron spectrometer (Shimadzu, Japan). 3D-Al and raw Al surface measurements were completed on a laser confocal microscope (Zeiss LSM 800). The contact angle was measured on a KRUSS GmbH DSA 1005 to analyse the wetting on the 3D-Al foil surface. The morphology and roughness of 3D-Al were observed using a multi-field infrared spectroscopy-atomic force microscopy coupled test system (Dimension ICON-IR). Metal concentrations were determined by full-spectrum direct-reading plasma emission spectrometer (Prodigy 7).

Electrochemical measurement

Swagelok® battery were assembled in an argon-filled glove box to evaluate the electrochemical performance of the porous Al foil. Symmetric cells were assembled using 3D-Al or pristine Al as cathode and anode in a 1.3:1 quaternary molten salt electrolyte separated by glass fibres (Whatman GF/AD grade, Sigma-Aldrich). The Al foils used in the symmetric cells were all 10 mm in diameter. The Al foils used for the symmetric cells were all 10 mm in diameter. Cyclic voltammetry (CV) and electrochemical impedance spectroscopy (EIS) were collected using a Bio-logic SP-200 potentiostat. The CV curves were scanned at a rate of 5 mVs^{-1}, and the EIS measurements covered the frequency range from 100 k Hz to 0.01 Hz with 85 data points. In addition, 3D-Al or pristine Al | GFD | graphite molten salt batteries were assembled in Swagelok® batteries using a 1.3:1 quaternary molten salt electrolyte. In detail, the cathode material was prepared by mixing graphite, Super P and polytetrafluoroethylene (PTFE) (8:1:1) in isopropanol, and the mixture was pressed into membranes having different mass loadings with a membrane cut-to-size mass of about 2-3 mg or so. Constant current charge/discharge measurements were performed using a battery test system (NEWARE). The charge rate capability of the cells was evaluated at different charge rates from 1 to 10 A. The CV curves were scanned at a rate of 5 mVs^{-1} and the EIS measurements covered the frequency range from 100 k Hz to 0.01 Hz. For freeze-thaw experiments, 3D-Al||graphite molten salt batteries were first tested for 30 cycles at a charge/discharge rate of 1 A g^{-1}. The temperature of the batteries was then brought down to room temperature by switching off the oven and cooling naturally to room temperature for 8-18 hours. Subsequently, the oven was started again to bring the temperature back to 90 °C.

DFT Calculations

In this study, all the spin-polarized DFT calculations were performed by using the Vienna Ab initio-Simulation Package (VASP). Pedew-Burke-Ermzerhof (PBE) functional in Generalized gradient approximation (GGA) was employed to describe the electron exchange-correlation interactions of the systems. The plane wave energy cutoff was set to 520 eV, and the energy and force convergence criteria were set to 1.0 $\times 10^{-5}$ eV per atom and -0.02 eV/Å, respectively. Structures were relaxed by using the conjugate gradient (CG) method. The k-mesh was sampling by the monkhorst-pack method with the value of 3*3*1. The weak and long-range interactions between adsorbates and substrate were described via the DFT-D3 method with BJ-damping.

Section S2 Results and discussion

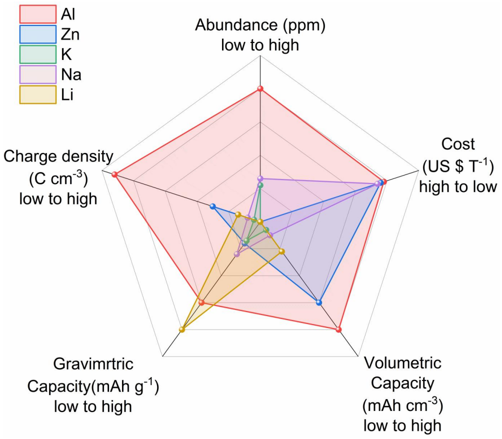

Figure S1. Comparisons of electrochemical properties, cost, and abundance for Al, Zn, K, Na, and Li.

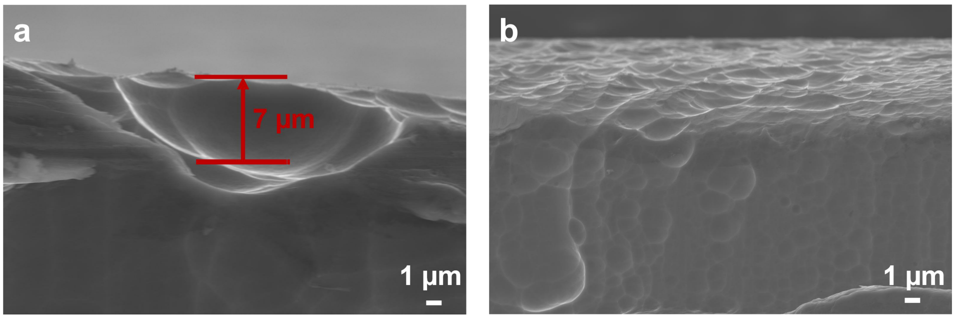

Figure S2. Cross-sectional SEM images of the 3D-Al.

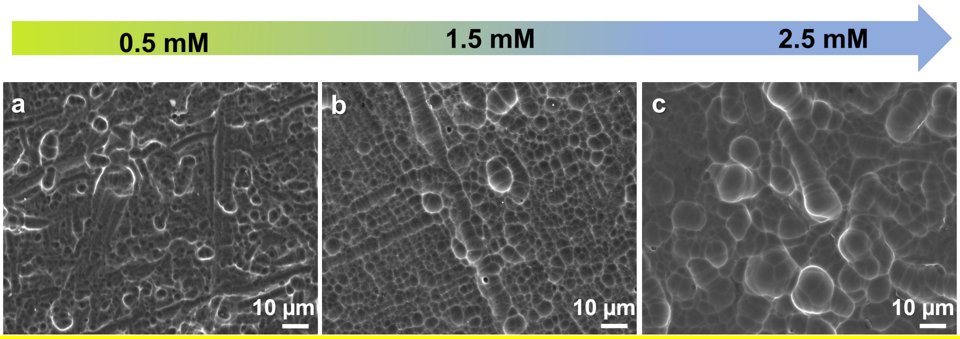

Figure S3. (a-c) SEM images of Al foil etched with different $Fe(NO_3)_3$ concentrations.

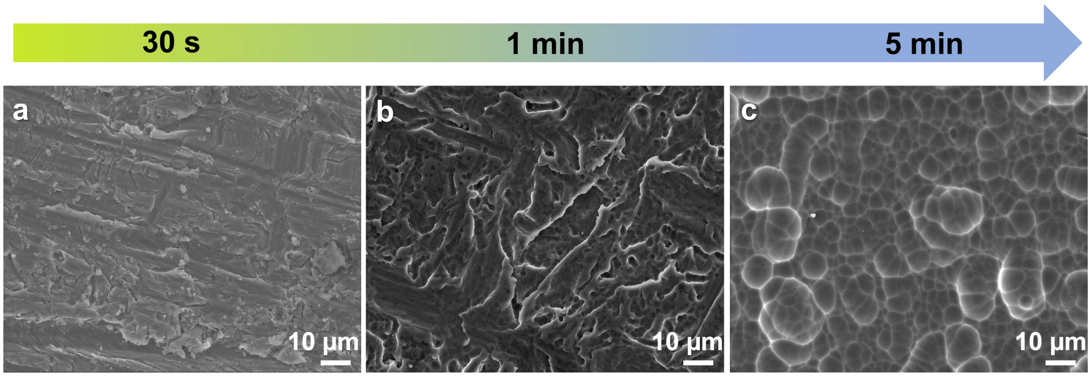

Figure S4. (a-c) SEM images of Al foil etched with the same concentration of $Fe(NO_3)_3$ for different times.

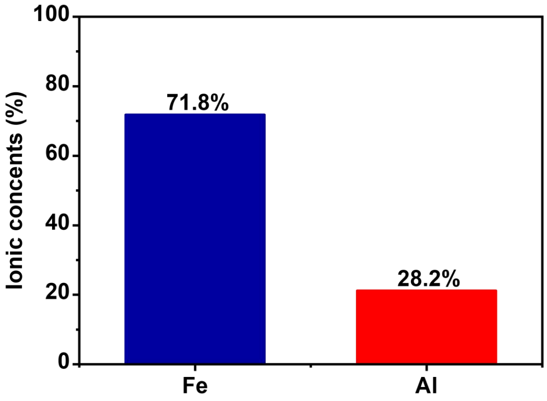

Figure S5. Al (III) ion contents of residual precursor solutions measured and calculated from the ICP results.

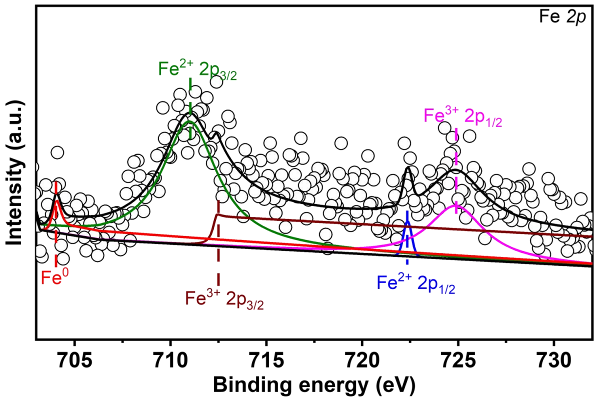

Figure S6. XPS spectra of Fe*2p* in 3D-Al.

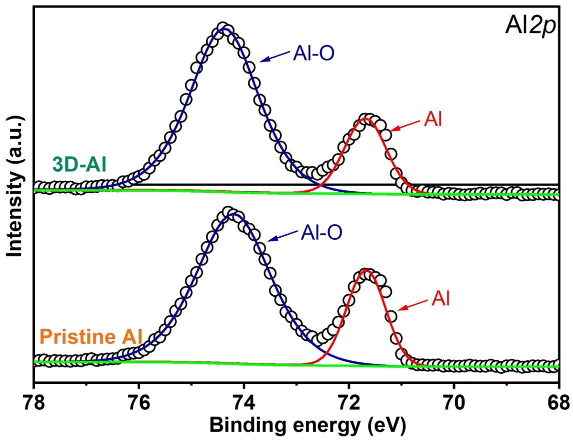

Figure S7. XPS spectra of Al*2p* in 3D-Al and pristine Al foils.

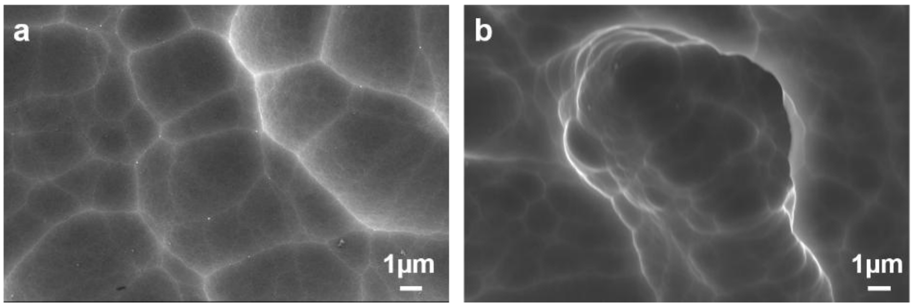

Figure S8. SEM images of the 3D-Al.

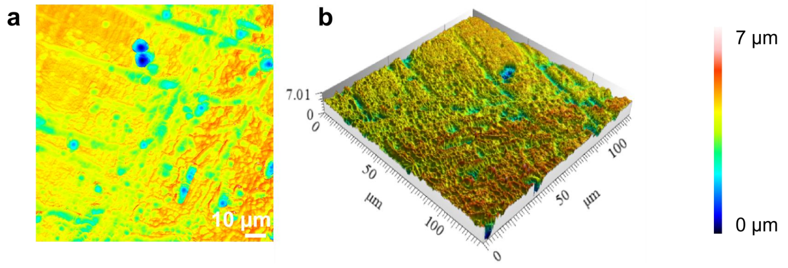

Figure S9. LSM images of the 3D-Al.

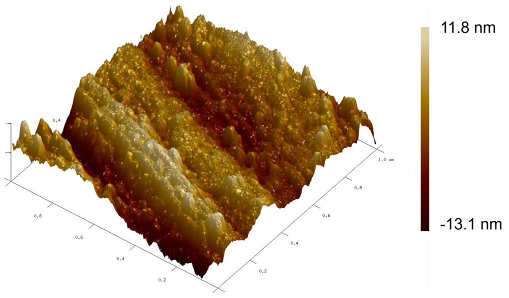

Figure S10. AFM images of the pristine Al foil.

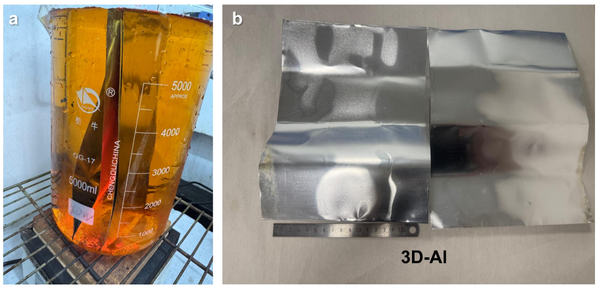

Figure S11. (a) Digital photo of the reaction equipment. (b) Digital photo of the large-scale preparation of the 3D-Al foils.

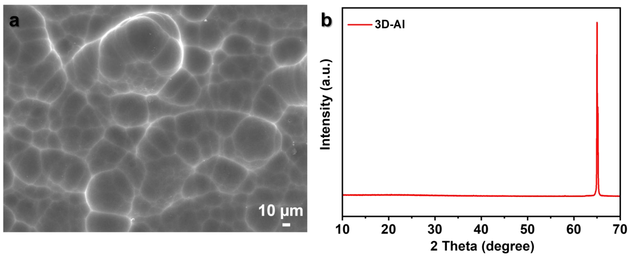

Figure S12. (a, b) SEM image and XRD pattern of the 3D-Al foil after large-scale preparation.

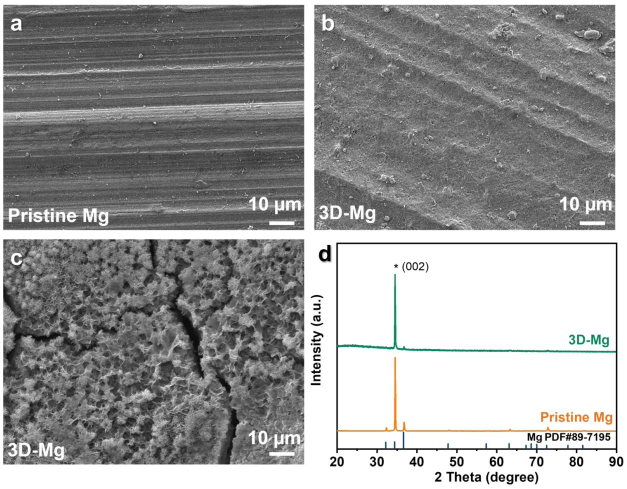

Figure S13. (a) SEM image of the pristine Mg. (b-c) SEM image of the 3D-Mg. (d) XRD patterns of pristine Mg and 3D-Mg.

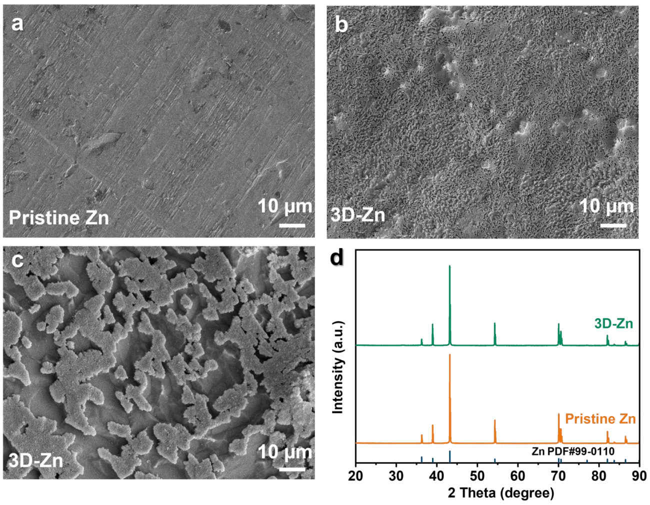

Figure S14. (a) SEM image of the pristine Zn. (b-c) SEM image of the 3D- Zn. (d) XRD patterns of pristine Zn and 3D- Zn.

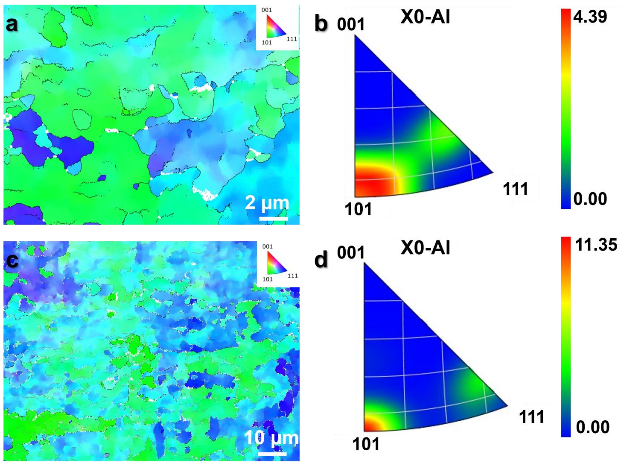

Figure S15. (a, b) EBSD orientation distribution map and corresponding antipolar diagram of the 3D-Al. (c, d) EBSD orientation distribution map and corresponding antipolar diagram of the 3D-Al in a large field of view.

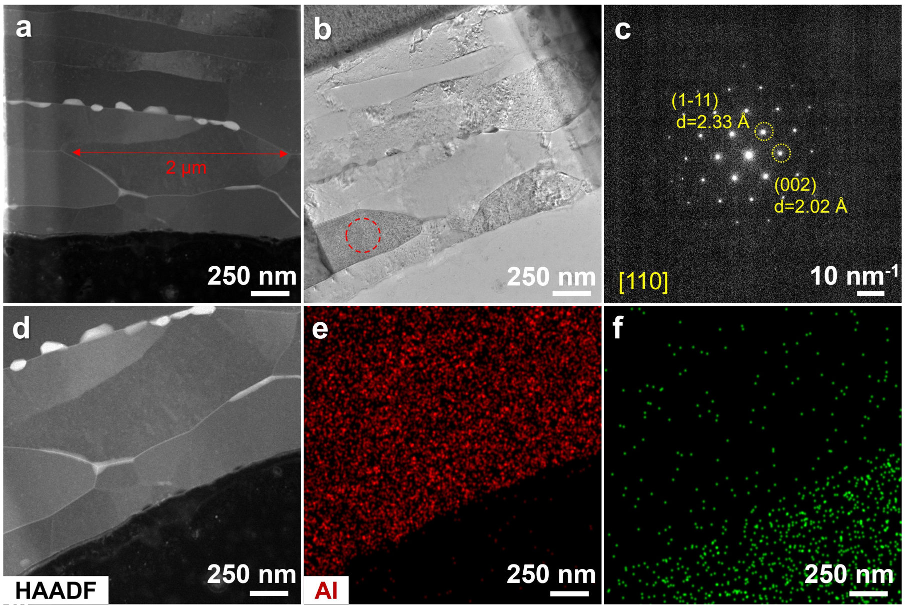

Figure S16. (a, b) Cross-sectional HAADF-STEM image and TEM image of the 3D-Al foil prepared by focused ion beam (FIB). (c) Fast Fourier transform (FFT) diffraction pattern of 3D-Al foil. (d) Cross-sectional HAADF-STEM images of the 3D-Al foil. (e, f) EDS elemental mapping images of Al and C elements corresponding to the FIB-prepared cross-section shown in (d).

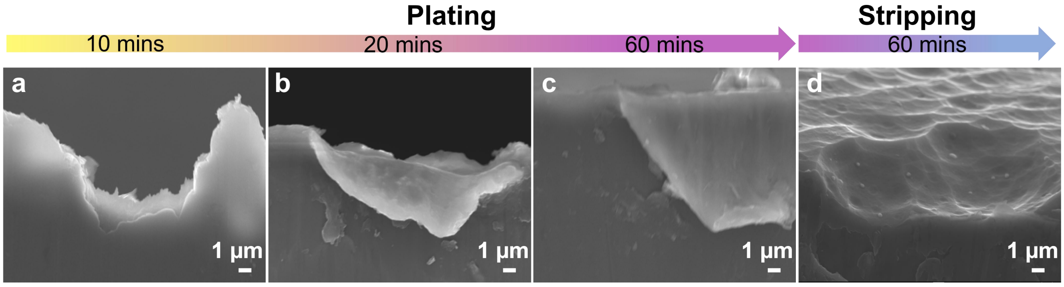

Figure S17. SEM morphology evolution of 3D-Al electrodes during plating/stripping for different durations at a current density of 0.5 mA cm^{-2}.

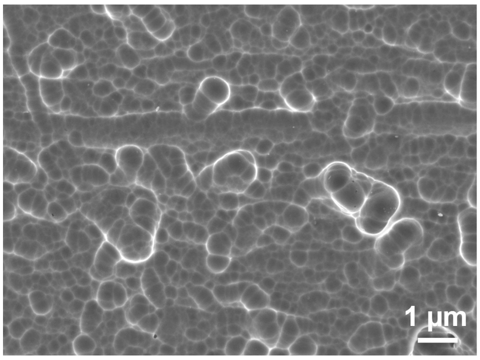

Figure S18. Low magnification SEM images of 3D-Al.

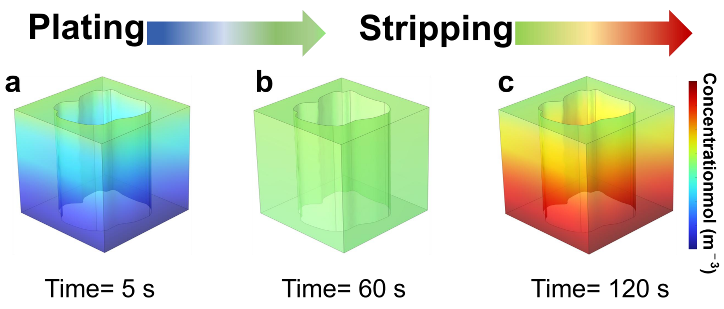

Figure S19. 3D COMSOL simulation of changes in Al^{3+} concentration and individual hole geometry during Al plating/stripping.

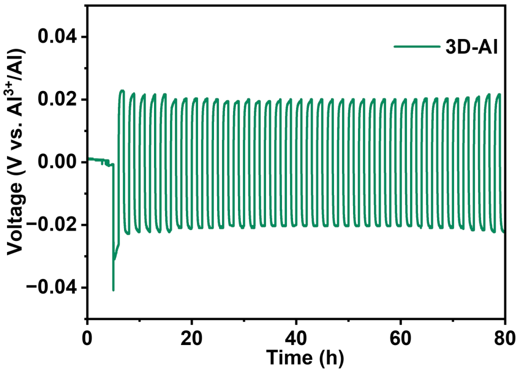

Figure S20. Charge-discharge curves of the 3D-Al based symmetric cell at a current density of 1 mA cm^{-2}.

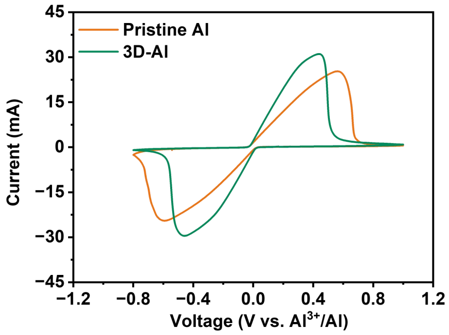

Figure S21. CV curves of 3D-Al and pristine Al based symmetric batteries at a scan rate of 5 mV s^{-1}.

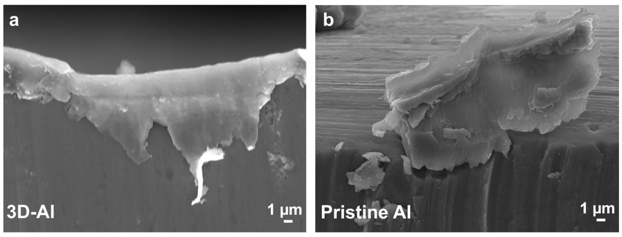

Figure S22. Cross-sectional SEM images of the 3D-Al (a) and pristine Al (b) based symmetric batteries after 5 cycles at a current density of 1 mA cm^{-2}.

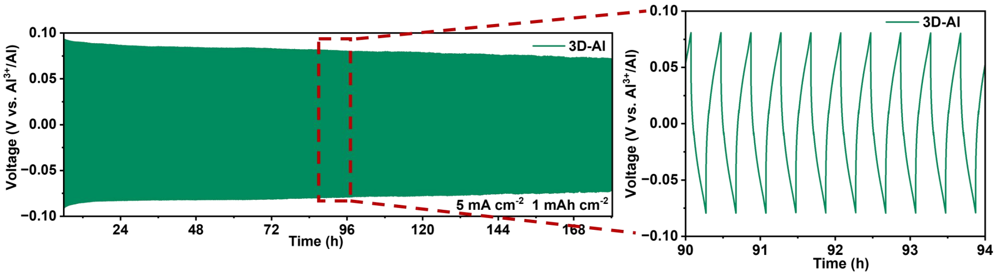

Figure S23. Long-term plating/stripping performance of 3D-Al and pristine Al based symmetric batteries at a current density of 5 mA cm^{-2}.

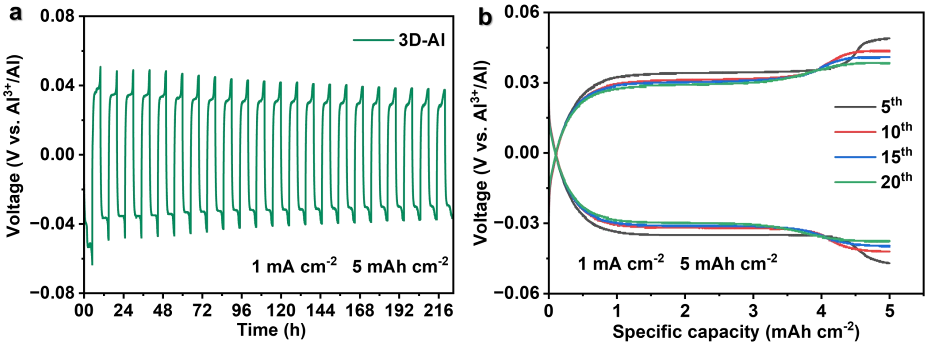

Figure S24. (a) Cycling performance of the 3D-Al symmetric cell at 1 mA cm^{-2}, 5 mAh cm^{-2} and (b) the corresponding voltage profiles.

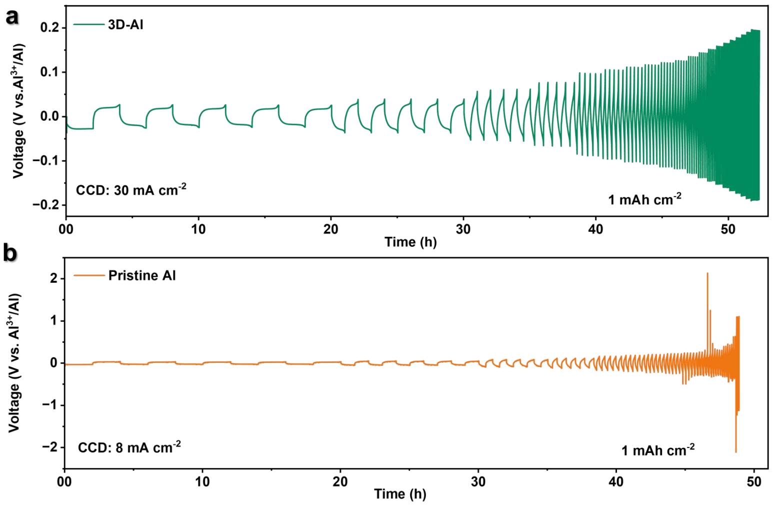

Figure S25. The voltage profiles of 3D-Al (a) and pristine Al (b) based symmetric cells within the capacity of 1 mAh cm^{-2} at different current densities (0.5, 1, 2, 3, 5, 6, 7, 8, 9, 10, 12, 14, 16, 18, 20, 22 24 26 28 and 30 mA cm^{-2}).

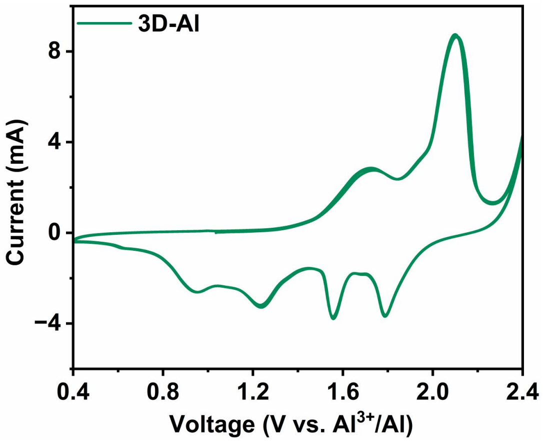

Figure S26. CV curves of 3D-Al||graphite and pristine Al||graphite molten salt batteries at a scan rate of 5 mV s^{-1}.

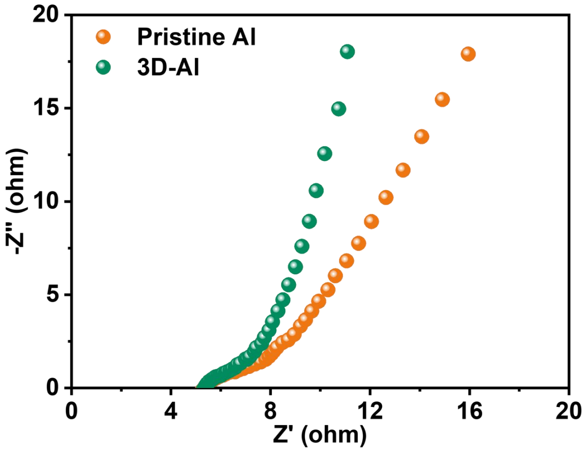

Figure S27. EIS spectra of the initial state of 3D-Al||graphite and pristine Al||graphite molten salt batteries.

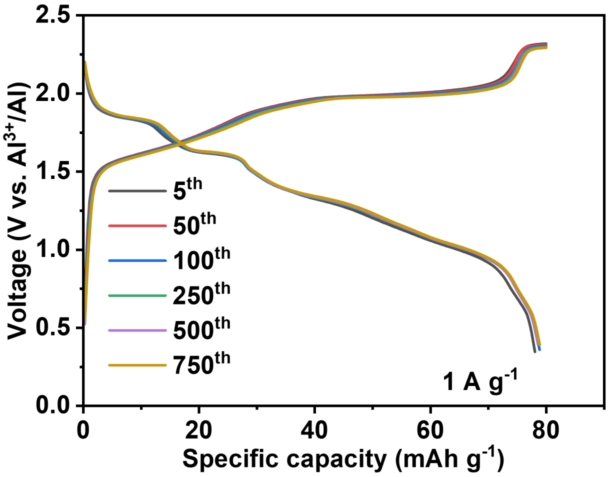

Figure S28. Voltage curves of the 3D-Al||graphite molten salt battery at 1 A g^{-1}.

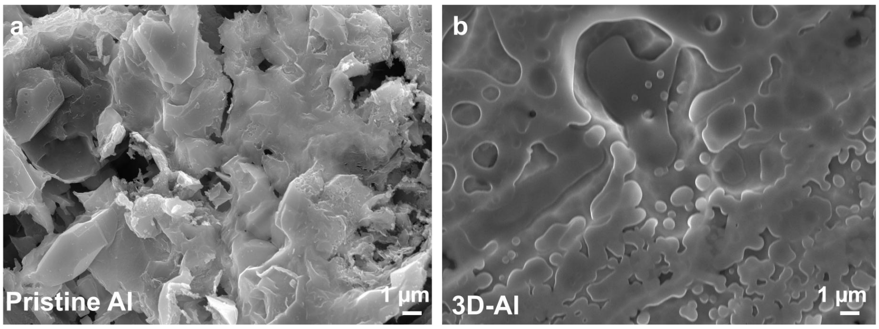

Figure S29. (a) SEM image of pristine Al in the molten salt battery after plating/stripping failure at a current density of 10 A g^{-1}. (b) SEM images of the 3D-Al in the molten salt battery after plating/stripping failure at a current density of 10 A g^{-1}.

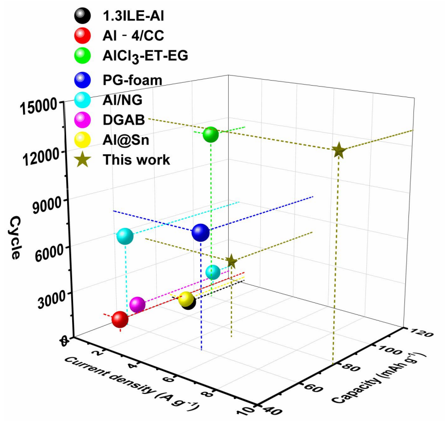

Figure S30. Comparison of the 3D-Al||graphite molten salt battery with previously reported works[1–7].

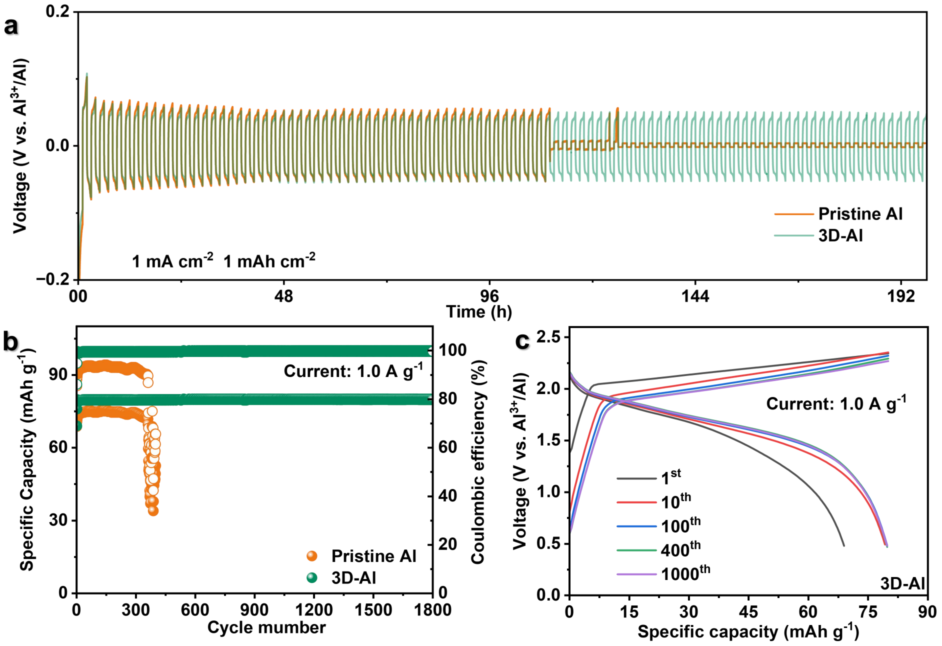

Figure S31. (a) Cycling performance of 3D-Al and pristine Al symmetric cells in ionic liquid electrolytes. (b) Cycling performance of 3D-Al||graphite and pristine

Al||graphite cells in ionic liquid electrolytes. (c) The corresponding voltage profiles of the 3D-Al||graphite cell.

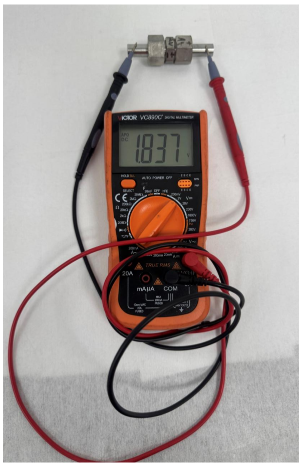

Figure S32. Open-circuit voltage of the 3D-Al||graphite molten salt battery.

Table S1. Results related to surface area, maximum depth, average depth and volume of 3D-Al based on LSM measurements.

Parametric	Unit	Hole	Peak
Surface	μm^2	64.8	4.88
Volume	μm^3	125	1.30
Maximum depth	μm	3.68	1.45
Average depth	μm	1.93	0.266

References

1. Lee D, Lee G, Tak Y. Hypostatic instability of aluminum anode in acidic ionic liquid for aluminum-ion battery. *Nanotechnology* 2018;**29**:36LT01.
2. Li J, Hui KS, Ji S *et al.* Electrodeposition of a dendrite-free 3D Al anode for improving cycling of an aluminum–graphite battery. *Carbon Energy* 2022;**4**:155–69.
3. Dong X, Xu H, Chen H *et al.* Commercial expanded graphite as high-performance cathode for low-cost aluminum-ion battery. *Carbon* 2019;**148**:134–40.
4. Lin M-C, Gong M, Lu B *et al.* An ultrafast rechargeable aluminium-ion battery. *Nature* 2015;**520**:324–8.
5. Wang D-Y, Wei C-Y, Lin M-C *et al.* Advanced rechargeable aluminium ion battery with a high-quality natural graphite cathode. *Nat Commun* 2017;**8**:14283.
6. Xie C, Wu F, Lv Z *et al.* A Mixed Ionic/Electronic Conductor Interphase Enhances Interfacial Stability for Aluminium-Metal Anode. *Adv Funct Mater*

2024;**34**:2408296.

7. Wang S, Jiao S, Song W-L *et al.* A novel dual-graphite aluminum-ion battery. *Energy Storage Mater* 2018;**12**:119–27.
